# Urinary Liver-Type Fatty-Acid-Binding Protein Predicts Long-Term Adverse Outcomes in Medical Cardiac Intensive Care Units

**DOI:** 10.3390/jcm9020482

**Published:** 2020-02-10

**Authors:** Hiroyuki Naruse, Junnichi Ishii, Hiroshi Takahashi, Fumihiko Kitagawa, Hideto Nishimura, Hideki Kawai, Takashi Muramatsu, Masahide Harada, Akira Yamada, Wakaya Fujiwara, Mutsuharu Hayashi, Sadako Motoyama, Masayoshi Sarai, Eiichi Watanabe, Hideo Izawa, Yukio Ozaki

**Affiliations:** 1 Department of Joint Research Laboratory of Clinical Medicine, Fujita Health University School of Medicine, Toyoake 470-1192, Japanfkitaga@fujita-hu.ac.jp (F.K.); 2 Division of Statistics, Fujita Health University School of Medicine, Toyoake 470-1192, Japan; hirotaka@fujita-hu.ac.jp; 3 Department of Cardiology, Fujita Health University School of Medicine, Toyoake 470-1192, Japanhidekikawai@xc4.so-net.ne.jp (H.K.); takam@fujita-hu.ac.jp (T.M.); mharada@fujita-hu.ac.jp (M.H.); a-yamada@fujita-hu.ac.jp (A.Y.); sadakom@fujita-hu.ac.jp (S.M.); msarai@fujita-hu.ac.jp (M.S.); enwatan@mtj.biglobe.ne.jp (E.W.);; 4 Department of Cardiology, Bantane Hospital, Nagoya 454-8509, Japan; wakayafj@fujita-hu.ac.jp (W.F.); muhayasi@med.nagoya-u.ac.jp (M.H.); izawa@fujita-hu.ac.jp (H.I.)

**Keywords:** liver-type fatty-acid-binding protein, long-term outcomes, cardiac intensive care units, acute kidney injury

## Abstract

We prospectively investigated the prognostic value of urinary liver-type fatty-acid-binding protein (L-FABP) levels on hospital admission, both independently and in combination with serum creatinine-defined acute kidney injury (AKI), to predict long-term adverse outcomes in 1119 heterogeneous patients (mean age; 68 years) treated at medical (non-surgical) cardiac intensive care units (CICUs). Patients with stage 5 chronic kidney disease were excluded from the study. Of these patients, 47% had acute coronary syndrome and 38% had acute decompensated heart failure. The creatinine-defined AKI was diagnosed according to the “Kidney Disease: Improving Global Outcomes” criteria. The primary endpoint was a composite of all-cause death or progression to end-stage kidney disease, indicating the initiation of maintenance dialysis therapy or kidney transplantation. Creatinine-defined AKI occurred in 207 patients, with 44 patients having stage 2 or 3 disease. During a mean follow-up period of 41 months after enrollment, the primary endpoint occurred in 242 patients. Multivariate Cox regression analyses revealed L-FABP levels as independent predictors of the primary endpoint (*p* < 0.001). Adding L-FABP to a baseline model with established risk factors further enhanced reclassification and discrimination beyond that of the baseline model alone, for primary-endpoint prediction (both; *p* < 0.01). On Kaplan–Meier analyses, increased L-FABP (≥4th quintile value of 9.0 ng/mL) on admission or presence of creatinine-defined AKI, correlated with an increased risk of the primary endpoint (*p* < 0.001). Thus, urinary L-FABP levels on admission are potent and independent predictors of long-term adverse outcomes, and they might improve the long-term risk stratification of patients admitted at medical CICUs, when used in combination with creatinine-defined AKI.

## 1. Introduction

Liver-type fatty-acid-binding protein (L-FABP; molecular weight, 14,000) is expressed in the proximal tubular epithelial cells [1] and binds to free fatty acids in the cytoplasm [2,3]. During renal injury, L-FABP binds to lipid peroxidation products and is excreted into urine, protecting the proximal tubules from oxidative stress [4]. Therefore, urinary L-FABP might be a suitable marker of renal tubular injury. In a heterogeneous cohort of patients treated in medical (nonsurgical) cardiac intensive care units (CICUs), urinary L-FABP levels were found to be potent predictors of acute kidney injury (AKI) [5,6]. However, to date, the association between urinary L-FABP levels and long-term adverse outcomes in patients treated at medical CICUs remains poorly understand.

The consensus definition of AKI is currently based on acute changes in serum creatinine levels or decreases in urine output. However, the serum creatinine-defined definition for AKI has several limitations because creatinine is a muscle metabolite that is an insensitive and nonspecific marker of kidney excretory function at only steady state [7]. Combining kidney excretory function parameter with tubular injury markers has prognostic relevance. Kidney tubular injury markers can predict the need for renal replacement, length of hospital stay, and in-hospital mortality in critically ill and surgical patients [8,9]. Furthermore, “subclinical AKI”, as evidenced by increased urinary tubular injury markers without serum creatinine increase, is associated with severe in-hospital clinical outcomes [10]. However, only few studies of patients after cardiac surgery have reported that tubular injury marker combined with creatinine-defined AKI status could predict the increased risk of long-term adverse outcomes [11,12].

This study prospectively investigated the prognostic value of urinary L-FABP levels on admission, both independently and in combination with creatinine-defined AKI, in patients hospitalized at medical CICUs.

## 2. Materials and Methods

### 2.1. Study Design

This prospective study was conducted at the Department of Cardiology, Fujita Health University School of Medicine (Toyoake, Japan). We enrolled patients hospitalized at medical (non-surgical) CICUs in Fujita Health University Hospital from January 2012 to December 2013. The ethics committee of Fujita Health University approved this study (study protocol number 11-053), which was in accordance with the Declaration of Helsinki. All patients individually provided written informed consent.

Patients with cardiovascular disease requiring hospitalization as determined by the attending physician of the medical CICUs were eligible for enrollment. We obtained urinary and blood samples upon admission, for baseline biomarker measurements. Meanwhile, we excluded patients who had the following characteristics—(1) under 18 years old, (2) undergoing cardiac surgery, (3) experiencing trauma, (4) having stage 5 chronic kidney disease (CKD), (5) receiving percutaneous cardiopulmonary support before admission, (6) having an active malignant disease being treated with chemotherapy or radiation, and (7) having autoimmune diseases. Independent physicians blinded to urinary L-FABP levels could freely select therapy as indicated. Clinical characteristics were obtained from patients’ medical records, upon enrollment.

### 2.2. Definitions and Calculations

Patients were diagnosed with serum creatinine-defined AKI, according to the “Kidney Disease: Improving Global Outcomes” criteria, that is, an increase in serum creatinine by ≥0.3 mg/dL, within 48 h, or an increase in serum creatinine to ≥1.5 times the baseline, within 1 week [13]. The lowest known serum creatinine value during the past 3 months was used as baseline creatinine. For patients with unknown baseline, we used the lowest creatinine value within 7 days after admission at medical CICUs. We calculated the serum creatinine-based estimated glomerular filtration rate (eGFR), using the Modification of Diet in Renal Disease Study equation, as recommended by the Japan CKD Initiative [14]. Incident end-stage kidney disease (ESKD) indicated the initiation of maintenance dialysis therapy or kidney transplantation, whereas an eGFR <60 mL/min/1.73 m^2^ indicated CKD.

Diabetes was defined as a history of or current diabetes or a fasting plasma glucose level ≥126 mg/dL, a hemoglobin A1c value ≥6.5%, or the presence of diabetic retinopathy. Hypertension was defined as having a systolic blood pressure ≥140 mmHg, a diastolic blood pressure ≥90 mmHg, or a history of antihypertensive treatment. Dyslipidemia was defined as a total cholesterol level ≥220 mg/dL or a history of lipid-lowering therapy. Patients with smoking history were classified as either current or ex-smokers. We calculated the sequential organ failure assessment (SOFA) score according to the worst value of the parameters, which include PaO_2_/FiO_2_, platelet count, bilirubin, mean arterial blood pressure and the use of vasoactive drugs, the Glasgow Coma Scale, and creatinine and urine output [15]. We routinely performed 2D echocardiography to calculate left ventricular ejection fraction (LVEF), using the modified Simpson’s method.

All patients were clinically followed up for a mean period of 41 months after enrollment. The primary endpoint was a composite of all-cause mortality or progression to ESKD. All-cause mortality was the secondary endpoint. Data for the endpoints were obtained from hospital charts and through telephone interviews with patients. The telephone interviews were conducted by trained reviewers who were blinded to the patient L-FABP levels.

### 2.3. Biomarker Measurements

Immediately after admission, urinary and blood samples were collected in nonheparinized tubes and then centrifuged at 1000× *g* at 4 °C for 15 min, before storage at −80 °C until assayed. We measured the urinary L-FABP levels by an enzyme-linked immunosorbent assay (ELISA) using the Human L-FABP ELISA Kit (CMIC, Tokyo, Japan). Plasma B-type natriuretic peptide (BNP) levels were measured using a chemiluminesence enzyme immunoassay for human BNP (Shionogi & Co., Ltd., Osaka, Japan). We measured serum high-sensitivity troponin T (hs-TnT) levels via an electrochemiluminescence immunoassay, using a Cobas® e601 system (Roche Diagnostics, Tokyo, Japan), and serum high-sensitivity C-reactive protein (hs-CRP) levels via a latex-enhanced hs-CRP immunoassay (N-Latex CRP II, Siemens Healthineers, Tokyo, Japan). Serum creatinine levels were determined by an enzyme method, using the Liquitech® Creatinine PAP II (Roche Diagnostics, Tokyo, Japan) upon admission, daily until day 3, and then on day 7.

### 2.4. Statistical Analyses

We performed statistical analyses using StatFlex version 6 (Artech Co. Ltd. Osaka, Japan). Normally distributed variables are expressed as mean values ± standard deviations, whereas nonparametric data are presented as medians and interquartile ranges. Considering that the urinary L-FABP, plasma BNP, serum hs-TnT, and serum hs-CRP data were irregularly distributed, analyses were performed after log-transformation to meet the criteria for use in normalized statistical approaches (after statistical confirmation). The relationship between urinary L-FABP and other baseline valuables was studied by linear regression analysis. Intergroup differences were evaluated by one-way analysis of variance or the Kruskal–Wallis test for continuous variables and by the chi-square test for categorical variables. Moreover, we examined the intergroup differences in endpoint by the Kaplan–Meier method and compared them using the log-rank test. Hazard ratios and 95% confidence intervals were calculated for each factor via the Cox proportional hazards analysis. All baseline variables with *p* < 0.05 in univariate analyses were integrated into the Cox multivariate model to determine the independent predictors of the endpoint.

To assess whether the accuracy of predicting the endpoint would improve after adding L-FABP into a baseline model with established risk factors, we calculated the C-index, net reclassification improvement (NRI), and integrated discrimination improvement (IDI). The established risk factors were as follows—age, hypertension, diabetes mellitus, CKD, paroxysmal or persistent atrial fibrillation, acute decompensated heart failure, myocardial infarction history, coronary revascularization history, systolic blood pressure, heart rate, mechanical ventilation before admission, and BNP. The C-index was defined as the area under the receiver operating characteristic curves between individual predictive probabilities for the endpoint and the incidence of endpoint, and it was compared with the baseline model [16]. NRI was a relative indicator of how many patients had improved in the predicted probability of the endpoint, whereas IDI indicated the average improvement in the predicted probability of the endpoint, after adding variables into the baseline model [17]. We considered *p* < 0.05 as statistically significant.

## 3. Results

### 3.1. Baseline Characteristics and Outcomes

We enrolled 1119 patients with a mean age of 68 years (23–83 years) and summarized their demographics and clinical characteristics in Table 1 and Table 2. These patients were admitted due to the following diagnoses—acute coronary syndrome (529 (47%)), acute decompensated heart failure (424 (38%)), arrhythmia (51 (5%)), primary pulmonary hypertension (32 (3%)), acute aortic syndrome (24 (2%)), infective endocarditis (14 (1%)), takotsubo cardiomyopathy (11 (1%)), and others (34 (3%)). Of these patients, 497 (44%) were diagnosed with CKD, with no case of kidney transplantation. Urinary L-FABP levels correlated with age (r = 0.06, *p* = 0.03), and eGFR (r = −0.25, *p* < 0.001). Among the 1119 patients, 207 (18.5%) developed creatinine-based AKI in which 44 had stage 2 or 3 disease.

During a mean follow-up period of 41 months, the primary endpoint occurred in 242 patients, of which 17 developed ESKD. All-cause death was manifested in 228 patients, of which 141 experienced cardiovascular deaths. Cardiovascular deaths were caused by heart failure in 85, myocardial infarction in 23, stroke in 17, sudden death in 10, and arrhythmia in 6 patients.

Patients who developed the primary endpoint were older and had higher SOFA score, higher heart rate, higher levels of glucose, hs-CRP, BNP, hs-TnT, and L-FABP, and lower levels of hemoglobin, eGFR, and LVEF than those who did not. Many patients who developed the primary endpoint had the following characteristics—coronary revascularization history; paroxysmal or persistent atrial fibrillation; acute decompensated heart failure; antiplatelet drugs; β blockers; diuretics; anticoagulant drugs; and AKI. The median length of medical CICUs stay in patients with primary endpoint (4.0 (3.0–6.0) days) was longer than those without (3.0 (2.0–4.0) days) (*p* < 0.001).

### 3.2. Prognostic Value of Urinary L-FABP

**Figure 1:**
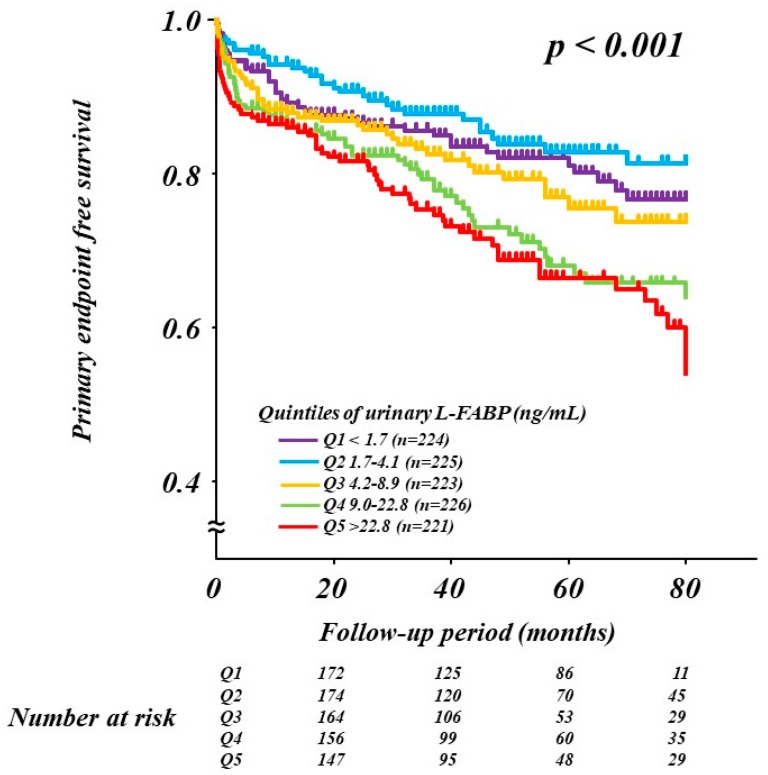
Kaplan–Meier curves for the primary endpoint according to the L-FABP level quintiles. L-FABP, liver-type fatty-acid-binding protein.

When patients were divided into quintiles according to the L-FABP levels (1st, <1.7 ng/mL; 2nd, 1.7–4.1 ng/mL; 3rd, 4.2–8.9 ng/mL; 4th, 9.0−22.8 ng/mL; and 5th, >22.8 ng/mL), the Kaplan–Meier primary-endpoint free survival rate in the 1st, 2nd, 3rd, 4th, and 5th L-FABP quintiles were 81.7%, 85.8%, 80.3%, 73.9%, and 70.1%, respectively (*p* < 0.001; Figure 1). Given that the Kaplan–Meier primary-endpoint free survival curves for the 1st, 2nd, and 3rd quintiles were relatively similar, as well as those for the 4th and 5th quintiles, we used the 4th quintile value for L-FABP (9.0 ng/mL) as the cutoff value for endpoint prediction.

As revealed in the Cox multivariate analysis, L-FABP was the independent predictor of the primary endpoint when assessed as either continuous variables (*p* < 0.001) or variables categorized by the 4th quintile value of 9.0 ng/mL (*p* < 0.001) (Table 3). Age, systolic blood pressure, hemoglobin, BNP, and LVEF also remained significantly associated with the primary endpoint. Similar results were obtained for all-cause mortality (Table 3).

### 3.3. Discrimination and Reclassification of L-FABP for Adverse Outcomes

We assessed the effect of adding L-FABP to a baseline model of established risk factors. As shown in Table 4, adding L-FABP significantly improved the reclassification of patients beyond that of the baseline model alone (*p* < 0.001); IDI improved similarly after adding L-FABP (*p* = 0.002). Conversely, the C-index did not improve beyond the baseline model alone, considering that the C-statistic is insensitive for comparing the models [18]. Similar results were seen for all-cause death (Table 4).

### 3.4. Combination of L-FABP and Creatinine-Defined AKI

**Figure 2:**
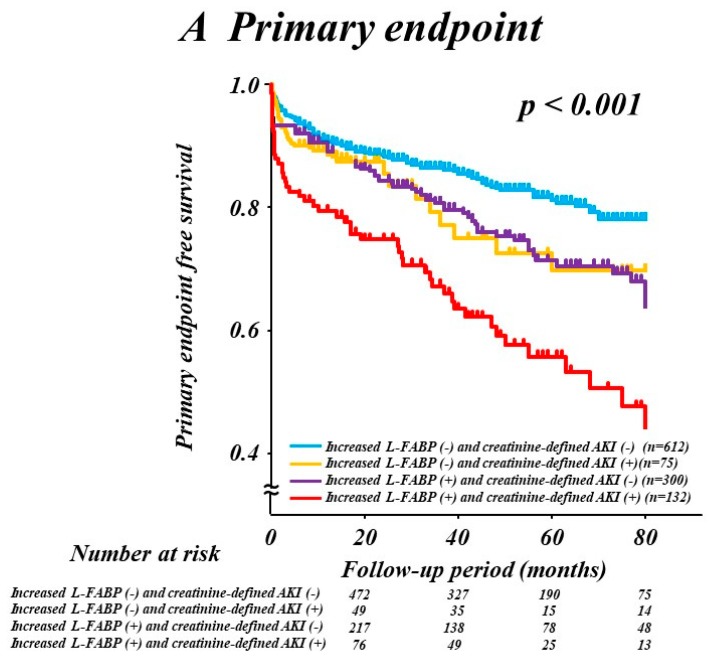

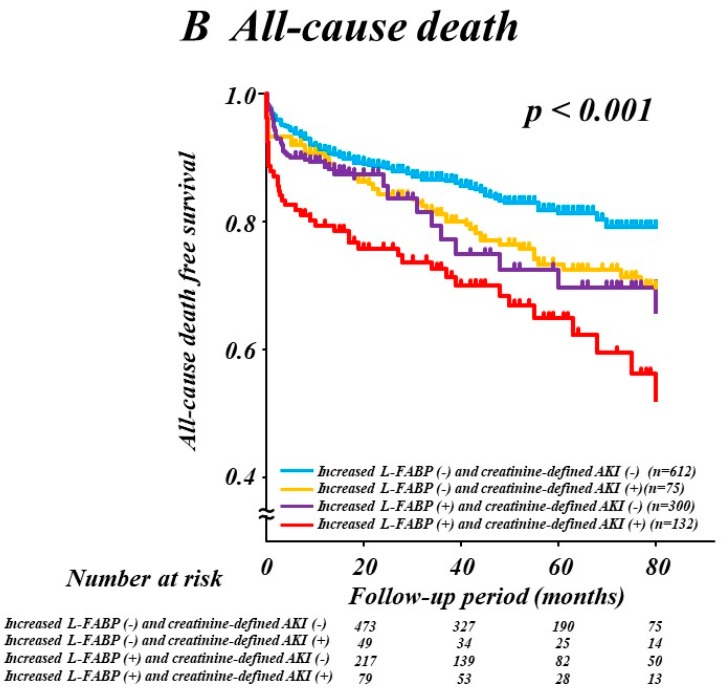
Kaplan–Meier curves for the primary endpoint (**A**) and all-cause mortality (**B**) according to L-FABP increment (≥9 ng/mL) or serum creatinine-defined AKI status. AKI, acute kidney injury; L-FABP, liver-type fatty-acid-binding protein.

We classified the patients into four groups according to the L-FABP increment (≥4th quintile value of 9.0 ng/mL) or creatinine-defined AKI status. The Kaplan–Meier primary-endpoint free survival rates were 83.5% in patients without increased L-FABP or creatinine-defined AKI (*n* = 612), 75.7% in patients without creatinine-defined AKI who had increased L-FABP (*n* = 300), 77.3% in patients with creatinine-defined AKI who did not have increased L-FABP (*n* = 75), and 61.4% in patients with both increased L-FABP and creatinine-defined AKI (*n* = 132) (*p* < 0.001; Figure 2). Increased L-FABP without creatinine-defined AKI was identified in 59% (*n* = 300) of patients with AKI, indicating subclinical AKI, compared with creatinine-defined AKI only, and these patients were at a greater risk of the primary endpoint than those without increased L-FABP or creatinine-defined AKI (*p* = 0.02). Similar results were observed for all-cause death (Figure 2).

## 4. Discussion

The prospective study obtained the following main findings. First, urinary L-FABP levels upon admission were significantly independent predictors of both the primary endpoint and all-cause mortality in patients treated at medical CICUs. Second, urinary L-FABP improved the predictive value for both the primary endpoint and all-cause mortality beyond that achieved with baseline model with established risk factors, as demonstrated by NRI and IDI. Third, the combination of increased L-FABP levels and creatinine-defined AKI status correlated with an increased risk of both the primary endpoint and all-cause mortality. Thus, urinary L-FABP levels on admission are potential and independent predictors of long-term adverse outcomes, and when used in combination with creatinine-defined AKI, they might improve the long-term risk stratification of patients hospitalized at medical CICUs. As supported by our results, the novel AKI definition that considers the urinary tubular injury biomarker concentrations, might be preferable to current definitions that are limited to changes in serum creatinine alone [7,10,11,12].

Although AKI is a common complication in patients treated at medical CICUs [19,20], urinary L-FABP levels in such patients has rarely been investigated, compared to those treated at ICUs after surgery (particularly cardiac) and those with septic patients [21,22]. The present study has demonstrated that urinary L-FABP on admission predicts long-term adverse outcomes in a large (*n* = 1119), heterogeneous cohort of patients treated at medical CICUs and that the combination of increased L-FABP and creatinine-defined AKI status improves the long-term risk stratification of patients hospitalized at medical CICUs.

Considering that urinary tubular injury markers provide information different from that provided by creatinine-defined AKI, the combined assessment of urinary tubular injury marker and creatinine-defined AKI status can be clinically beneficial. According to the present study, the combination of increased L-FABP on admission and the presence of creatinine-defined AKI could stratify the long-term prognostic risk of patients treated at medical CICUs, as shown in patients after cardiac surgery [21]. As expected, severe long-term outcomes were found in patients with both increased L-FABP and creatinine-defined AKI (tubular damage with kidney excretory dysfunction), whereas favorable outcomes were observed in patients without increased L-FABP or creatinine-defined AKI (no tubular damage and no excretory dysfunction). Urinary L-FABP identified approximately 60% more patients with increased L-FABP but without creatinine-defined AKI (tubular damage without excretory dysfunction)—an indicator of subclinical AKI—than those with creatinine-defined AKI status alone. This outcome was intermediate in severity. A smaller group (6.7%) of patients had creatinine-defined AKI but no increased L-FABP, implying the loss of renal function without the evidence of acute tubular injury and showing an intermediate outcome. These findings are consistent with recent reports involving patients after cardiac surgery, suggesting that the urinary tubular marker complements creatinine-defined AKI in long-term prognosis [11,12].

Urinary renal tubular injury markers have been investigated in critically ill patients, for an earlier identification of AKI, improved AKI diagnosis, and aid in risk stratification [9,22,23,24]. However, the association of urinary tubular injury markers with long-term mortality has been seldom studied [11,12]. In the Translation Research Investigating Biomarker Endpoints for Acute Kidney Injury (TRIBE-AKI) study involving 1,199 adult patients who underwent cardiac surgery, Coca et al. showed that higher urinary interleukin 18 (IL-18) and kidney injury molecule 1 (KIM-1) levels were independently associated with 3-year mortality, regardless of the creatinine-defined AKI status [11]. Moreover, in 200 adult patients who also underwent cardiac surgery, Albert et al. reported that the combined assessment of urinary neutrophil gelatinase-associated lipocalin (NGAL) with creatinine-defined AKI status could stratify the risk of mortality within a median follow-up period of 5.6 years [12]. The authors also confirmed an increased risk of long-term mortality in subclinical AKI patients. These two studies did not evaluate BNP and high-sensitivity troponin levels, markers of left ventricular overload and myocardial injury, respectively, even though these markers are also prognostic markers in patients with cardiovascular disease. Thus, urinary L-FABP levels were independent predictors in the multivariate Cox regression model of clinical and laboratory parameters including BNP, in a heterogeneous cohort of 1119 patients treated at medical CICUs. Given that the hs-TnT levels were not significantly associated with long-term adverse outcomes in a univariate Cox regression analysis, it was not integrated in the Cox multivariate model. Furthermore, adding urinary L-FABP improved the predictive value for long-term adverse outcomes beyond that achieved with the baseline model of established risk factors, including BNP, as demonstrated by NRI and IDI.

The mechanisms that emphasize the association between increased L-FABP and long-term adverse outcomes are still unclear. Kidneys are an excellent barometer of cardiac and vascular function [25]. Recently, in 968 adult post-cardiac surgery patients from the TRIBE-AKI cohort, Parikh et al. found that the plasma markers for cardiac injury (high-sensitivity troponin and heart-type fatty-acid-binding protein) or left ventricular overload (N-terminal pro-BNP), but not urinary tubular injury markers (IL-18, NGAL, KIM-1, L-FABP, and albumin), were independently associated with increased long-term risk of cardiovascular events [25]. Thus, higher urinary L-FABP levels might reflect a more severe AKI but might not be on the casual pathway to long-term adverse outcomes. Patients who manifest a more severe AKI might be at a greater risk for long-term complications, such as death, caused by a worse functioning of other organs rather than the episode of AKI [11].

In the present study, we used ELISA, which requires approximately 3 hours to measure the urinary L-FABP levels. Recently, a latex-enhanced immunoturbidimetric assay has been established using an autoanalyzer, which was simple, speedy (within 30 min) [26], and relatively inexpensive to assay, for quantifying urinary L-FABP. Thus, the easy and rapid assay system is expected to facilitate urinary L-FABP analysis in clinical practice.

Our study had several limitations. First, this study was conducted at a single institution. Second, we evaluated urinary L-FABP as an absolute concentration; we did not use urinary creatinine correction because the urinary creatinine excretion rate might change over time under nonsteady state conditions [27]. When the L-FABP values were analyzed using urinary creatinine correction, no association was found between the L-FABP levels and outcomes (data not shown). Finally, AKI was only defined according to serum creatinine increase, because of the inconsistent data recorded and the potential alterations in urine volume induced by medical therapy. This limitation might lead to the neglect of a part of the renal insult, which might be determined by urine output.

## 5. Conclusions

Urinary L-FABP levels on admission are potent and independent predictors of long-term adverse outcomes in patients treated at medical CICUs. When used in combination with creatinine-defined AKI, urinary L-FABP might substantially improve the long-term risk stratification of patients admitted at medical CICUs.

## Figures and Tables

**Table 1:** Primary diagnosis.

Acute coronary syndrome, *n* (%)	529 (47)
STEMI, *n*	217
NSTEM, *n*	264
Unstable angina, *n*	48
Acute decompensated heart failure, *n* (%)	424 (38)
With reduced ejection fraction (LVEF < 40%), *n*	217
With mid-range ejection fraction (40% ≤ LVEF < 50%), *n*	67
With preserved ejection fraction (LVEF ≥ 50%), *n*	140
Arrhythmia, *n* (%)	51 (5)
Supraventricular tachycardia, *n*	6
Ventricular tachycardia, *n*	14
Sick sinus syndrome, *n*	13
Second- or third-degree atrioventricular block, *n*	18
Primary pulmonary hypertension, *n* (%)	32 (3)
Acute aortic syndrome, *n* (%)	24 (2)
Infective endocarditis, *n* (%)	14 (1)
Takotsubo cardiomyopathy, *n* (%)	11 (1)
Others, *n* (%)	34 (3)

Data are expressed as numbers (%). STEMI, ST-segment elevation myocardial infarction; NSTEMI, non-ST-segment elevation myocardial infarction; LVEF, left ventricular ejection fraction.

**Table 2:** Baseline characteristics of study population according to primary endpoint.

	All Patients	Primary Endpoint (+)	Primary Endpoint (-)	*p* Value
Number	1119	242	877	
Age (year)	68 ± 12	73 ± 9	67 ± 13	<0.001
Male, *n* (%)	732 (65)	157 (65)	575 (66)	0.84
Hypertension, *n* (%)	724 (65)	158 (65)	566 (65)	0.83
Dyslipidemia, *n* (%)	520 (47)	97 (40)	423 (48)	0.02
Diabetes, *n* (%)	420 (38)	88 (36)	332 (38)	0.67
Current or ex-smoker, *n* (%)	324 (29)	70 (29)	254 (29)	0.99
Previous myocardial infarction, *n* (%)	214 (19)	61 (25)	153 (17)	0.007
Prior hospitalization for worsening heart failure, *n* (%)	215 (19)	53 (22)	162 (19)	0.23
Previous coronary revascularization, *n* (%)	213 (19)	59 (24)	154 (18)	0.02
Paroxysmal or persistent AF, *n* (%)	248 (22)	77 (32)	171 (20)	<0.001
Acute decompensated heart failure, *n* (%)	424 (38)	143 (59)	281 (32)	<0.001
SOFA score	2 (1–4)	4 (2–5)	2 (1–4)	<0.001
Systolic blood pressure, mmHg	141 ± 31	135 ± 32	143 ± 31	<0.001
Heart rate, beats per minutes	86 ± 25	90 ± 24	85 ± 26	0.001
Emergent CAG or PCI before admission, *n* (%)	405 (36)	69 (29)	336 (38)	0.005
Mechanical ventilation before admission, *n* (%)	20 (1.8)	6 (2.5)	14 (1.6)	0.36
IABP before admission, *n* (%)	96 (8.6)	20 (8.3)	76 (8.7)	0.84
White blood cell count, ×10^3^/μL	8.7 ± 3.6	8.4 ± 3.9	8.7 ± 3.4	0.19
Hemoglobin, g/dL	12.7 ± 2.3	11.7 ± 2.3	13.0 ± 2.2	<0.001
eGFR, mL/min/1.73 m^2^	66.6 ± 26.6	54.2 ± 25.2	70.0 ± 26.0	<0.001
Glucose, mg/dL	159 ± 70	170 ± 75	156 ± 68	0.006
hs-CRP, mg/L	2.32 (0.75–10.3)	4.50 (1.09–24.3)	1.99 (0.69–8.18)	<0.001
BNP, pg/mL	186 (53–631)	581 (158–1210)	133 (43–479)	<0.001
hs-TnT, pg/mL	59 (17–445)	56 (24–290)	62 (15–51)	0.43
Urinary L-FABP, ng/mL	5.8 (2.4–16.9)	9.2 (3.1–27.0)	5.2 (2.2–14.5)	<0.001
LVEF, %	47.3 ± 13.8	42.4 ± 14.4	48.7 ± 13.3	<0.001
Treatment at enrollment, *n* (%)				
Antiplatelet drugs	387 (35)	111 (46)	276 (32)	<0.001
Statins	355 (32)	70 (29)	285 (33)	0.29
RAAS inhibitors	469 (42)	110 (46)	359 (41)	0.21
Beta-blockers	301 (27)	84 (35)	217 (25)	0.002
Diuretics	305 (27)	103 (43)	202 (23)	<0.001
Anticoagulant drugs	163 (15)	52 (22)	111 (13)	<0.001
Creatinine-defined AKI, *n* (%)	207 (18.5)	68 (28.1)	139 (15.8)	<0.001

Data are presented as number (%), mean ± standard deviation, or median (25th–75th percentile). AF, atrial fibrillation; SOFA, Sequential Organ Failure Assessment; CAG, coronary angiography; PCI, percutaneous coronary intervention; IABP, intra-aortic balloon pump; eGFR, creatinine-based estimated glomerular filtration rate; hs-CRP, high-sensitivity C-reactive protein; BNP, B-type natriuretic peptide; hs-TnT, high-sensitivity cardiac troponin T; L-FABP, liver-type fatty-acid-binding protein; LVEF, left ventricular ejection fraction; RAAS, renin–angiotensin–aldosterone system; AKI, acute kidney injury.

**Table 3:** Multivariate predictors of primary endpoint and all-cause mortality.

**(A) Primary Endpoint**	**Model 1**		**Model 2**	
**Variables**	**HR (95% CI)**	***p* Value**	**HR (95% CI)**	***p* Value**
Age (per 10 years increment)	1.54 (1.32–1.81)	<0.001	1.54 (1.32–1.80)	<0.001
Previous myocardial infarction	0.81 (0.56–1.18)	0.27	0.86 (0.59–1.25)	0.43
Paroxysmal or persistent AF	1.16 (0.87–1.55)	0.32	1.16 (0.87–1.56)	0.31
Previous coronary revascularization	1.09 (0.75–1.59)	0.66	1.06 (0.73–1.55)	0.76
Acute decompensated heart failure	1.03 (0.74–1.42)	0.87	1.06 (0.77–1.47)	0.72
Systolic blood pressure (per 10 mmHg increment)	0.94 (0.90–0.98)	0.004	0.93 (0.89–0.97)	0.002
Heart rate (per 10 beats per minutes increment)	1.03 (0.98–1.08)	0.26	1.03 (0.98–1.09)	0.26
Hemoglobin (per 1 g/dL increment)	0.88 (0.83–0.94)	<0.001	0.88 (0.83–0.94)	<0.001
CKD	1.14 (0.85–1.54)	0.38	1.18 (0.88–1.58)	0.28
hs-CRP (per 10-fold increment)	1.08 (0.91–1.27)	0.40	1.09 (0.93–1.29)	0.29
BNP (per 10-fold increment)	1.84 (1.37–2.49)	<0.001	1.80 (1.34–2.44)	<0.001
Urinary L-FABP (per 10-fold increment)	1.47 (1.22–1.76)	<0.001		
Urinary L-FABP (ng/mL)				
< 9.0 (1st + 2nd + 3rd quintile)			Reference	
≥ 9.0 (4th + 5th quintile)			1.63 (1.25–2.12)	<0.001
LVEF (per 10% increment)	0.86 (0.78–0.96)	0.005	0.87 (0.79–0.97)	0.01
**(B) All-cause Mortality**	**Model 1**		**Model 2**	
**Variables**	**HR (95% CI)**	***p* Value**	**HR (95% CI)**	***p* Value**
Age (per 10 years increment)	1.66 (1.41–1.96)	<0.001	1.66 (1.40–1.96)	<0.001
Previous myocardial infarction	0.85 (0.58–1.24)	0.40	0.89 (0.61–1.31)	0.56
Paroxysmal or persistent AF	1.20 (0.89–1.61)	0.24	1.20 (0.89–1.62)	0.24
Previous coronary revascularization	1.11 (0.75–1.63)	0.60	1.08 (0.73–1.59)	0.70
Acute decompensated heart failure	1.00 (0.71–1.39)	0.99	1.02 (0.73–1.43)	0.90
Systolic blood pressure (per 10 mmHg increment)	0.93 (0.89–0.97)	0.002	0.92 (0.88–0.97)	<0.001
Heart rate (per 10 beats per minutes increment)	1.03 (0.97–1.08)	0.35	1.02 (0.97–1.08)	0.37
Hemoglobin (per 1 g/dL increment)	0.90 (0.85–0.96)	0.002	0.91 (0.85–0.97)	0.004
CKD	1.02 (0.75–1.37)	0.92	1.05 (0.78–1.42)	0.74
hs-CRP (per 10-fold increment)	1.13 (0.95–1.35)	0.17	1.15 (0.97–1.37)	0.10
BNP (per 10-fold increment)	1.89 (1.39–2.57)	<0.001	1.86 (1.36–2.53)	<0.001
Urinary L-FABP (per 10-fold increment)	1.43 (1.18–1.72)	<0.001		
Urinary L-FABP (ng/mL)				
< 9.0 (1st + 2nd + 3rd quintile)			Reference	
≥ 9.0 (4th + 5th quintile)			1.50 (1.14–1.97)	0.003
LVEF (per 10% increment)	0.87 (0.78–0.97)	0.009	0.88 (0.79–0.98)	0.02

Multivariate model adjusted for all baseline variables with *p* < 0.05 by univariate analysis. L-FABP levels were assessed as either continuous variables (Model 1) or variables categorized into quintiles (Model 2). HR, hazard ratio; CI, confidence interval; AF, atrial fibrillation; CKD, chronic kidney disease; hs-CRP, high-sensitivity C-reactive protein; BNP, B-type natriuretic peptide; L-FABP, liver-type fatty-acid-binding protein; LVEF, left ventricular ejection fraction.

**Table 4:** Discrimination and reclassification of L-FABP.

**(A) Primary Endpoint**						
	**C-index**	***p* Value**	**NRI**	***p* Value**	**IDI**	***p* Value**
Established risk factor model	0.756	Reference		Reference		Reference
Established risk factor model + L-FABP	0.763	0.76	0.252	<0.001	0.013	0.002
**(B) All-cause Mortality**						
	**C-index**	***p* Value**	**NRI**	***p* Value**	**IDI**	***p* Value**
Established risk factor model	0.760	Reference		Reference		Reference
Established risk factor model + L-FABP	0.766	0.80	0.222	0.001	0.012	0.004

Established risk factors included age, hypertension, diabetes mellitus, chronic kidney disease, atrial fibrillation, acute decompensated heart failure, previous myocardial infarction, previous coronary revascularization, systolic blood pressure, heart rate, mechanical ventilation before admission, and B-type natriuretic peptide. L-FABP, liver-type fatty-acid-binding protein; NRI, net reclassification improvement; IDI, integrated discrimination improvement.
